# Medication use and contextual factors associated with meeting guideline-based glycemic levels in diabetes among a nationally representative sample

**DOI:** 10.3389/fmed.2023.1158454

**Published:** 2023-05-31

**Authors:** Cassidi C. McDaniel, Wei-Hsuan Lo-Ciganic, Kimberly B. Garza, Jan Kavookjian, Brent I. Fox, Chiahung Chou

**Affiliations:** ^1^Department of Health Outcomes Research and Policy, Harrison College of Pharmacy, Auburn University, Auburn, AL, United States; ^2^Department of Pharmaceutical Outcomes & Policy, College of Pharmacy, University of Florida, Gainesville, FL, United States; ^3^Center for Drug Evaluation and Safety, College of Pharmacy, University of Florida, Gainesville, FL, United States; ^4^Department of Medical Research, China Medical University Hospital, Taichung City, Taiwan

**Keywords:** glycemic control, diabetes management, A1C, medication use, contextual factors

## Abstract

**Introduction:**

Based on the long-lasting diabetes management challenges in the United States, the objective was to examine glycemic levels among a nationally representative sample of people with diabetes stratified by prescribed antihyperglycemic treatment regimens and contextual factors.

**Methods:**

This serial cross-sectional study used United States population-based data from the 2015 to March 2020 National Health and Nutrition Examination Surveys (NHANES). The study included non-pregnant adults (≥20 years old) with non-missing A1C and self-reported diabetes diagnosis from NHANES. Using A1C lab values, we dichotomized the outcome of glycemic levels into <7% versus ≥7% (meeting vs. not meeting guideline-based glycemic levels, respectively). We stratified the outcome by antihyperglycemic medication use and contextual factors (e.g., race/ethnicity, gender, chronic conditions, diet, healthcare utilization, insurance, etc.) and performed multivariable logistic regression analyses.

**Results:**

The 2042 adults with diabetes had a mean age of 60.63 (SE = 0.50), 55.26% (95% CI = 51.39–59.09) were male, and 51.82% (95% CI = 47.11–56.51) met guideline-based glycemic levels. Contextual factors associated with meeting guideline-based glycemic levels included reporting an “excellent” versus “poor” diet (aOR = 4.21, 95% CI = 1.92–9.25) and having no family history of diabetes (aOR = 1.43, 95% CI = 1.03–1.98). Contextual factors associated with lower odds of meeting guideline-based glycemic levels included taking insulin (aOR = 0.16, 95% CI = 0.10–0.26), taking metformin (aOR = 0.66, 95% CI = 0.46–0.96), less frequent healthcare utilization [e.g., none vs. ≥4 times/year (aOR = 0.51, 95% CI = 0.27–0.96)], being uninsured (aOR = 0.51, 95% CI = 0.33–0.79), etc.

**Discussion:**

Meeting guideline-based glycemic levels was associated with medication use (taking vs. not taking respective antihyperglycemic medication classes) and contextual factors. The timely, population-based estimates can inform national efforts to optimize diabetes management.

## 1. Introduction

The United States diabetes prevalence has accumulated significantly over time since 1999 (1), reaching the most recent estimate of 14.7% (37.1 million adults) (2). Diabetes management is challenging and complex (routine disease monitoring, medications, healthy lifestyle behaviors, patient education, and periodic healthcare visits) (3). Despite these challenges, adequate disease management is necessary to meet the guideline-based glycemic levels. Meeting guideline-based glycemic levels is critical because chronically unmanaged diabetes is associated with increased microvascular and macrovascular complications (4). Achieving guideline-based glycemic levels in diabetes reduces the likelihood of developing these complications (5–7).

The American Diabetes Association’s (ADA) Standards of Care in Diabetes recommend achieving an A1C < 7% to avoid diabetes-related complications (8, 9). Still, the prevalence of high glycemic levels in diabetes has remained a concern for decades. Nearly half of the people with diabetes had an A1C ≥ 7% from 1999 to 2018 in the United States (10, 11). The prevalence of A1C ≥ 7% has remained relatively stable, with a high of 56% in 1999–2002 and a low of 43% in 2007–2010 (10). Thus, identifying the factors affecting glycemic levels can inform target intervention strategies for diabetes management.

Previous studies identified various influential factors for glycemic levels in diabetes. For instance, glycemic levels varied based on different antihyperglycemic medication use (10, 12, 13). Other factors influencing glycemic levels include age (14), race (15), employment (15), diabetes duration (14), comorbidities (12), diabetes complications (13), and receipt of annual A1C checks (14). The Translating Research Into Action for Diabetes (TRIAD) study found young age, female gender, non-White race, low educational status, high body mass index (BMI), smoking behavior, low physical activity, and challenges with the cost of care to be significantly associated with “poor control” in diabetes (16). However, the TRIAD study included glycemic levels, blood pressure, and cholesterol in defining “poor control” (16). Further research should investigate meeting guideline-based glycemic levels in diabetes and generate findings with high external validity for the United States.

These prior works highlight the need to investigate prescribed antihyperglycemic medications and additional factors in understanding glycemic levels. We sought to provide adjusted, population-based estimates unavailable in the current literature by examining a holistic range of factors (broadly referred to as contextual factors) that could potentially influence glycemic levels. We defined contextual factors following the TRIAD conceptual model, which outlines the relationships between diabetes-related outcomes with “fixed patient factors,” “clinical and psychosocial factors,” “patient-physician-system interactions,” “care processes,” and “behaviors” (17). Our objective was to examine glycemic levels (represented by A1C) among a nationally representative sample of people with diabetes stratified by their prescribed antihyperglycemic treatment regimens and contextual factors.

## 2. Materials and methods

### 2.1. Study design

We conducted a cross-sectional study by analyzing a retrospective, large-scale, nationally representative survey database. The Institutional Review Board at the author’s institution approved the study through an exempt application.

### 2.2. Theoretical framework

The Andersen’s Behavioral Model, first developed in the 1960s, theoretically informed this study based on the contribution of behaviors on diabetes-related outcomes (18). The Andersen’s Behavioral Model received several updates over time, with the most recent updates demonstrating contextual factors’ direct and indirect influence on health outcomes (18). The Andersen’s Behavioral Model outlines how contextual and individual factors influence people’s health behaviors and subsequent outcomes (18). The Andersen’s Behavioral Model supports our investigation of glycemic levels (i.e., the health outcome) by studying potential associations with contextual factors, individual factors, and health behaviors (discussed further in Section 2.6) (18).

### 2.3. Data source

The National Health and Nutrition Examination Survey (NHANES) data are publicly available and were retrospectively collected from participants through health interviews at one time point (not longitudinally) (19). The NHANES typically releases data in two-year cycles. However, the COVID-19 pandemic precluded the completion of the 2019–2020 cycle, so the NHANES created the 2017-March 2020 Pre-pandemic cycle to ensure nationally representative estimates (20). We lumped the two most recently released data cycles (NHANES 2015–2016 and NHANES 2017-March 2020 Pre-pandemic) into one cohort for analysis. The NHANES provides relevant information for this study, including A1C lab values to assess glycemic levels, self-reported medications, and various contextual factors collected through demographic, nutritional, examination, laboratory, and questionnaire data. The NHANES also enabled the calculation of population-based estimates.

### 2.4. Study population and sample

The NHANES recruited a nationally representative sample of United States adults with diabetes to complete health interviews and examinations (19). We restricted the sample to include non-pregnant adults (≥20 years old based on the NHANES’ sampling definition) with non-missing A1C values (0.04% of people with diabetes had missing A1C) and a self-reported diabetes diagnosis (diabetes types unavailable in the NHANES data).

### 2.5. Outcome measures

The primary outcome was meeting vs. not meeting guideline-based glycemic levels (A1C < 7% vs. A1C ≥ 7%, respectively). Our categorization of glycemic levels followed the ADA 2023 Standards of Care in Diabetes, where A1C < 7% is the guideline-based glycemic goal recommended for the general, non-pregnant adult population (8).

The ADA Standards of Care in Diabetes also acknowledge the need for individualized glycemic goals, such as less stringent glycemic goals among people with older age or more complications (8, 21). We performed a sensitivity analysis applying similar methods as Kazemian et al. to calculate individualized glycemic goals (22). We classified respondents as meeting individualized glycemic levels at A1C < 7% for adults <65 years old with <three chronic conditions, A1C < 7.5% for adults <65 years old with ≥three chronic conditions, A1C < 7.5% for adults ≥65 years old with <three chronic conditions, and A1C < 8% for adults ≥65 years old with ≥three chronic conditions (21, 22). We used a cut-off of three chronic conditions to identify individualized glycemic goals following the ADA’s Standards (21). We included the self-reported chronic conditions listed in Supplementary Table S1 and retinopathy or kidney problems to capture diabetes-related complications (22).

### 2.6. Covariates

We selected covariates for inclusion following the TRIAD conceptual model (16, 17), the Andersen’s Behavioral Model (18), and prior research (10, 12, 13). The TRIAD conceptual model directly informed our conceptualization of covariates influencing glycemic levels and our categorization of covariates into the following constructs: “fixed patient factors,” “clinical and psychosocial factors,” “patient-physician-system interactions,” “care processes,” and “behaviors” (17). The Andersen’s Behavioral Model was used as a foundational theoretical framework to support our assumption that various factors/covariates could influence glycemic levels (18). We stratified glycemic levels by the key variables: antihyperglycemic medication class (Supplementary Table S2) and number. We investigated self-reported antihyperglycemic medications from the Prescription Medications Questionnaire of the NHANES data. We created dichotomous medication class variables (1 = taking medication in this class vs. 0 = not taking) following the respective medication classes outlined in Multum’s Lexicon database (23) and the ADA guidelines (24). A respondent coded as 0 for one medication class could still be taking an antihyperglycemic medication from a different class. These medication class categories were also not mutually exclusive because a respondent could simultaneously take antihyperglycemic medications from zero, one, or more classes. We also adjusted for contextual factors informed by the TRIAD conceptual model (Supplementary Table S1) (16, 17) and calendar year.

### 2.7. Statistical analysis

We used the survey procedures available in SAS to ensure population-based estimates while incorporating the complex survey design of the NHANES data (i.e., accounting for weight, strata, and cluster variables) (25). We converted the original cycle weights to 5.2-year cycle weights when combining the NHANES cycles (25). We calculated variance estimations using Taylor series linearization methods as the National Center for Health Statistics recommended (25). We completed analyses using SAS version 9.4 (SAS Institute, Cary, NC).

Our analytic sample had no missing data for the outcome measure based on the exclusion criteria discussed in Section 2.4 above. The analytic sample also had no missing data for the variables representing prescribed antihyperglycemic regimens because we classified respondents that did not report any prescribed antihyperglycemic medications as taking zero prescribed antihyperglycemic medications. Approximately 31% (*N* = 625, unweighted) of the sample had missing data for any covariates. To handle potential nonresponse bias, we imputed missing data for these covariates using weighted random hot deck imputation (26). We chose this imputation method because of its popular use for survey nonresponse and the use of survey sampling weights to select donors (26). We imputed missing data using responses from survey respondents in the same dataset, where respondents were similar to non-respondents according to the following specified adjustment cells (26): gender, race/ethnicity, and age group (categorized as 20–39, 40–49, 50–54, 55–59, 60–64, 65–69, 70–74, 75–80).

We provided descriptive statistics for characteristics among the overall sample and by glycemic levels. In unadjusted analyses, we used chi-square tests and two-sample *t*-tests as appropriate to investigate antihyperglycemic treatment regimens and contextual factors. We set an *a priori* level of significance at 0.05 and used two-sided hypothesis testing.

We used multivariable logistic regression models in adjusted analyses to identify characteristics associated with meeting guideline-based glycemic levels (i.e., A1C < 7%) after adjusting for confounders. We ran a full regression model that included all covariates, and we also used stepwise selection to identify a more parsimonious model. We applied the stepwise selection approach previously developed by Wang and Shin (27) to maintain the complex survey design and produce population-based estimates. We used a conservative of *p*-value 0.10 for variables entry and exit during model building. We reported adjusted odds ratios (aOR) and 95% confidence intervals (95% CI). We repeated the multivariable logistic regression analyses in the sensitivity analysis to identify characteristics associated with meeting individualized glycemic levels.

## 3. Results

### 3.1. Sample characteristics and guideline-based glycemic levels

Among the 2042 respondents with diabetes in the NHANES 2015-March 2020 Pre-pandemic, 51.82% (95% CI = 47.11–56.51; *N* = 969) met guideline-based glycemic levels (A1C < 7%). When using individualized glycemic goals, 63.08% (95% CI = 59.13–66.90; *N* = 1,178) met guideline-based glycemic levels. Table 1 shows the characteristics of the overall sample and stratified by glycemic levels. The overall sample had a mean age of 60.63 (SE = 0.50) years, was 55.26% male (95% CI = 51.39–59.09), and was 64.49% (95% CI = 59.19–69.53) non-Hispanic White/other race/ethnicity.

**Table 1:** Characteristics of people with diabetes in NHANES 2015–2020 (pre-pandemic), by glycemic level (A1C).

Characteristics	Total Sample^a^ *N* = 2042 Wt. % (95% CI)	A1C < 7% *N* = 969 Wt. % (95% CI)	A1C ≥ 7% *N* = 1,073 Wt. % (95% CI)	*p*-value
**Fixed patient factors**				
Age, mean (SE)	60.63 (0.50)	61.32 (0.72)	59.89 (0.57)	0.09
*Race/ethnicity*				
Non-Hispanic White/other	64.49 (59.19–69.53)	68.83 (64.19–73.20)	59.82 (51.21–68.00)	0.009
Hispanic	16.84 (13.24–20.97)	13.90 (10.83–17.44)	20.01 (14.52–26.48)	
Non-Hispanic Black	12.90 (9.86–16.46)	11.71 (8.96–14.96)	14.17 (10.03–19.22)	
Non-Hispanic Asian	5.77 (4.20–7.70)	5.56 (3.90–7.65)	6.00 (4.10–8.43)	
*Gender*				
Male	55.26 (51.39–59.09)	51.28 (45.82–56.72)	59.54 (55.32–63.66)	0.004
Female	44.74 (40.91–48.61)	48.72 (43.28–54.18)	40.46 (36.34–44.68)	
*Family history of diabetes*				
Yes	73.15 (70.35–75.81)	69.66 (65.18–73.88)	76.89 (72.98–80.49)	0.02
No	26.85 (24.19–29.65)	30.34 (26.12–34.82)	23.11 (19.51–27.02)	
*Family monthly poverty level index*				
≤1.30	23.41 (20.41–26.61)	21.02 (17.42–24.98)	25.98 (21.73–30.58)	0.14
1.30 < index ≤1.85	14.11 (11.96–16.48)	14.01 (11.38–16.98)	14.22 (11.10–17.83)	
>1.85	62.49 (58.83–66.04)	64.98 (60.63–69.14)	59.81 (53.85–65.55)	
*Marital status*				
Married/living with partner	65.65 (61.78–69.38)	65.78 (61.20–70.15)	65.51 (60.41–70.37)	0.76
Widowed/divorced/separated	25.11 (21.53–28.95)	24.49 (20.24–29.16)	25.77 (21.76–30.10)	
Never married	9.24 (7.48–11.25)	9.72 (7.51–12.33)	8.72 (6.13–11.95)	
*Education level*				
Less than high school	17.89 (15.28–20.74)	16.34 (13.06–20.06)	19.55 (16.34–23.10)	0.19
High school graduate/GED	28.14 (24.65–31.84)	26.34 (22.54–30.41)	30.09 (24.77–35.83)	
Some college/associate degree	32.24 (28.98–35.64)	33.38 (27.57–39.58)	31.02 (26.81–35.49)	
College graduate or above	21.73 (18.36–25.40)	23.95 (19.03–29.44)	19.34 (15.83–23.25)	
*Occupation type*				
Looking for work or with a job/business but not at work	3.18 (2.14–4.53)	3.11 (1.52–5.59)	3.25 (2.14–4.72)	0.12
Not working at a job/business	54.59 (50.55–58.59)	57.95 (51.96–63.78)	50.98 (45.77–56.17)	
Working at a job/business for work	42.23 (38.70–45.82)	38.94 (34.22–43.82)	45.77 (40.14–51.48)	
*Acculturation: language spoken at home*				
English only	78.84 (74.42–82.81)	80.64 (75.08–85.43)	76.90 (71.71–81.54)	0.18
Non-English only	10.87 (8.65–13.43)	10.31 (7.39–13.89)	11.47 (8.96–14.40)	
English and non-English	10.29 (7.96–13.03)	9.05 (6.48–12.22)	11.63 (8.82–14.96)	
*Household food security* *^b^*				
Full food security	67.30 (64.08–70.41)	71.39 (67.69–74.88)	62.92 (58.73–66.96)	<0.001
Marginal food security	12.74 (10.10–15.77)	10.28 (8.13–12.77)	15.38 (11.74–19.62)	
Low food security	12.39 (10.37–14.65)	11.82 (9.42–14.58)	13.01 (10.33–16.08)	
Very low food security	7.56 (6.09–9.26)	6.51 (4.99–8.33)	8.69 (6.76–10.97)	
**Prescribed antihyperglycemic treatment regimens**				
*Class of prescribed antihyperglycemic medication*				
*Insulins*				
Yes	26.55 (24.14–29.07)	14.37 (10.80–18.58)	39.66 (35.77–43.64)	<0.001
No	73.45 (70.93–75.86)	85.63 (81.42–89.20)	60.34 (56.36–64.23)	
*Metformin*				
Yes	55.60 (52.80–58.37)	55.31 (50.49–60.07)	55.91 (51.58–60.17)	0.86
No	44.40 (41.63–47.20)	44.69 (39.93–49.51)	44.09 (39.83–48.42)	
*Sulfonylureas*				
Yes	23.66 (20.67–26.85)	16.41 (12.60–20.84)	31.45 (27.40–35.71)	<0.001
No	76.34 (73.15–79.33)	83.59 (79.16–87.40)	68.55 (64.29–72.60)	
*TZD*				
Yes	3.56 (2.26–5.31)	4.21 (2.11–7.43)	2.86 (1.42–5.09)	0.38
No	96.44 (94.69–97.74)	95.79 (92.57–97.89)	97.14 (94.91–98.58)	
*DPP-4i*				
Yes	8.05 (5.99–10.53)	5.80 (3.84–8.36)	10.46 (6.51–15.69)	0.07
No	91.95 (89.47–94.01)	94.20 (91.64–96.16)	89.54 (84.31–93.49)	
*SGLT-2i*				
Yes	4.27 (2.58–6.61)	4.37 (1.77–8.81)	4.17 (2.78–5.98)	0.90
No	95.73 (93.39–97.42)	95.63 (91.19–98.23)	95.83 (94.02–97.22)	
*GLP-1 RA*				
Yes	5.16 (3.67–7.03)	4.37 (2.11–7.90)	6.01 (3.87–8.85)	0.39
No	94.84 (92.97–96.33)	95.63 (92.10–97.89)	93.99 (91.15–96.13)	
*Combinations*				
Yes	5.63 (3.89–7.84)	4.21 (2.61–6.39)	7.16 (4.09–11.49)	0.14
No	94.37 (92.16–96.11)	95.79 (93.61–97.39)	92.84 (88.51–95.91)	
*Others*				
Yes	1.82 (0.99–3.05)	1.91 (0.76–3.91)	1.73 (0.79–3.27)	0.83
No	98.18 (96.95–99.01)	98.09 (96.09–99.24)	98.27 (96.73–99.21)	
Number of prescribed antihyperglycemic medications (continuous 0–5), mean (SE)	1.50 (0.03)	1.21 (0.06)	1.81 (0.05)	<0.001
**Clinical and psychosocial factors**				
Depressive symptoms (PHQ-9 score), mean (SE)	3.79 (0.15)	3.88 (0.21)	3.70 (0.20)	0.52
Duration of diabetes in years, mean (SE)	12.32 (0.41)	11.60 (0.57)	13.10 (0.42)	0.007
Body mass index (kg/m^2^), mean (SE)	32.90 (0.27)	32.68 (0.29)	33.14 (0.39)	0.29
Systolic blood pressure (mmHg), mean (SE)	128.83 (0.70)	127.99 (0.92)	129.74 (0.77)	0.09
Diastolic blood pressure (mmHg), mean (SE)	71.61 (0.39)	71.21 (0.44)	72.04 (0.58)	0.23
Total cholesterol (mg/dL), mean (SE)	174.33 (1.96)	172.46 (2.82)	176.35 (2.46)	0.28
Average sleep hours per night during weekdays, mean (SE)	7.63 (0.06)	7.67 (0.08)	7.59 (0.08)	0.42
Total number of chronic conditions, mean (SE)	1.80 (0.07)	1.93 (0.09)	1.66 (0.10)	0.03
**Behaviors**				
*Frequency of blood glucose self-monitoring*				
Never	23.19 (20.53–26.03)	29.98 (25.32–34.97)	15.89 (12.99–19.16)	<0.001
Multiple times daily	24.84 (22.24–27.59)	17.29 (14.08–20.89)	32.97 (28.11–38.11)	
Once daily	20.67 (17.55–24.07)	19.71 (15.75–24.16)	21.70 (17.20–26.78)	
Weekly (once or more)	20.35 (17.20–23.81)	20.52 (16.82–24.64)	20.17 (15.03–26.16)	
Less than weekly	10.94 (8.89–13.28)	12.50 (9.18–16.49)	9.26 (6.78–12.29)	
*Frequency of feet self-monitoring*				
Never	19.20 (16.29–22.38)	21.11 (17.60–24.97)	17.14 (13.30–21.56)	0.23
Daily (once or more)	49.49 (46.22–52.76)	47.40 (42.21–52.63)	51.73 (46.98–56.46)	
Weekly (once or more)	20.90 (18.25–23.76)	22.36 (17.88–27.36)	19.33 (16.34–22.61)	
Less than weekly	10.41 (7.82–13.52)	9.13 (6.36–12.60)	11.79 (7.68–17.08)	
*Self-reported healthiness of overall diet*				
Excellent	6.24 (4.71–8.07)	7.32 (4.89–10.46)	5.06 (3.27–7.45)	<0.001
Very good	17.60 (15.48–19.88)	21.26 (17.54–25.37)	13.66 (10.25–17.69)	
Good	44.83 (41.44–48.25)	44.94 (39.59–50.37)	44.71 (40.91–48.55)	
Fair	24.55 (22.06–27.17)	21.52 (18.47–24.82)	27.80 (24.19–31.64)	
Poor	6.79 (5.13–8.79)	4.96 (3.08–7.50)	8.76 (6.75–11.15)	
*Smoking status*				
Never smoked	48.50 (44.50–52.52)	47.47 (41.40–53.59)	49.61 (45.27–53.96)	0.81
Former smoker	36.40 (32.28–40.67)	37.29 (31.07–43.84)	35.44 (31.13–39.93)	
Current smoker	15.10 (12.76–17.68)	15.24 (11.58–19.52)	14.95 (12.21–18.03)	
*Physical activity: MET minutes per week*				
Physical activity not reported	32.68 (29.52–35.97)	31.40 (28.13–34.81)	34.06 (29.27–39.11)	0.16
Does not meet recommendation (<450)	27.32 (24.26–30.55)	29.06 (24.85–33.55)	25.45 (22.48–28.60)	
Meets recommendation (450 ≤ MET ≤750)	13.06 (11.50–14.73)	14.70 (12.12–17.60)	11.28 (8.34–14.82)	
Exceeds recommendation (>750)	26.94 (23.73–30.34)	24.84 (20.74–29.30)	29.20 (23.59–35.33)	
**Care processes**				
*A1C test within past year*				
Yes	89.50 (87.45–91.32)	89.09 (86.16–91.58)	89.94 (86.61–92.68)	0.67
No	10.50 (8.68–12.55)	10.91 (8.42–13.84)	10.06 (7.32–13.39)	
*Foot exam from doctor within past year*				
Yes	73.96 (70.78–76.97)	70.68 (66.85–74.32)	77.49 (72.66–81.83)	0.01
No	26.04 (23.03–29.22)	29.32 (25.68–33.15)	22.51 (18.17–27.34)	
*Most recent eye exam (dilated pupils)*				
Within the past year	66.89 (63.32–70.32)	67.09 (61.33–72.49)	66.67 (60.93–72.06)	0.98
Over 1 year ago	28.33 (25.33–31.47)	28.00 (23.01–33.44)	28.67 (23.65–34.12)	
Never	4.79 (3.58–6.25)	4.91 (2.93–7.65)	4.66 (3.10–6.70)	
**Patient-physician-system interactions**				
*Routine place for healthcare use*				
Yes	94.64 (92.68–96.20)	95.59 (93.35–97.24)	93.61 (90.57–95.91)	0.16
No	5.36 (3.80–7.32)	4.41 (2.76–6.65)	6.39 (4.09–9.43)	
*Frequency of healthcare use over past year*				
≥4 times	61.30 (58.49–64.05)	64.61 (59.80–69.21)	57.74 (53.27–62.11)	0.14
2 to 3 times	28.80 (26.24–31.46)	26.87 (22.59–31.48)	30.87 (27.36–34.56)	
Once	6.80 (5.24–8.65)	5.77 (4.12–7.82)	7.90 (5.11–11.57)	
None	3.11 (2.26–4.16)	2.76 (1.73–4.15)	3.49 (2.30–5.05)	
*Insurance coverage type*				
Private insurance	32.52 (28.77–36.45)	30.83 (24.93–37.23)	34.33 (28.33–40.73)	0.06
Medicare	11.12 (9.15–13.34)	11.50 (8.91–14.52)	10.71 (8.01–13.94)	
Medicaid	5.22 (4.02–6.65)	4.23 (2.58–6.50)	6.28 (4.42–8.63)	
Others	7.47 (5.88–9.34)	7.75 (5.37–10.76)	7.18 (5.45–9.25)	
Multiple plans	36.30 (33.04–39.64)	40.19 (34.59–45.98)	32.11 (28.10–36.32)	
Uninsured	7.38 (5.84–9.17)	5.50 (4.15–7.13)	9.39 (6.55–12.94)	
*Out-of-pocket prescription costs covered by insurance*				
Insurance does cover prescriptions	87.76 (85.41–89.85)	89.41 (86.62–91.79)	85.98 (82.10–89.29)	0.06
Insurance does not cover prescription	5.50 (3.99–7.36)	5.57 (3.41–8.52)	5.42 (3.72–7.58)	
Uninsured	6.75 (5.34–8.39)	5.02 (3.73–6.58)	8.61 (6.04–11.81)	
*NHANES cycle*				
2015–2016	36.93 (33.27–40.71)	36.12 (30.56–41.97)	37.81 (32.30–43.55)	0.68
2017-March 2020 pre-pandemic	63.07 (59.29–66.73)	63.88 (58.03–69.44)	62.19 (56.45–67.70)	

a Missing data for covariates were present for approximately 31% (N = 625, unweighted) of the sample, and missing data for these covariates were imputed using weighted random hot deck imputation with the following specified adjustment cells: gender, race/ethnicity, and age group. The population-based percentages included imputed data, so the percentages add up to equal 100%.

b NHANES assessed survey respondents’ food security using the United States Food Security Survey Module questions. NHANES created the categories of “full food security,” “marginal food security,” “low food security,” and “very low food security” based on the respondents’ number of “affirmative responses” (28).

TZD, thiazolidinediones; DPP-4i, dipeptidyl peptidase 4 inhibitors; SGLT-2i, sodium-glucose cotransporter-2 inhibitors; GLP-1 RA, glucagon-like peptide 1 receptor agonists; PHQ-9, Patient Health Questionnaire-9; MET, metabolic equivalent.

### 3.2. Glycemic levels by prescribed antihyperglycemic treatment regimens

**Figure 1:**
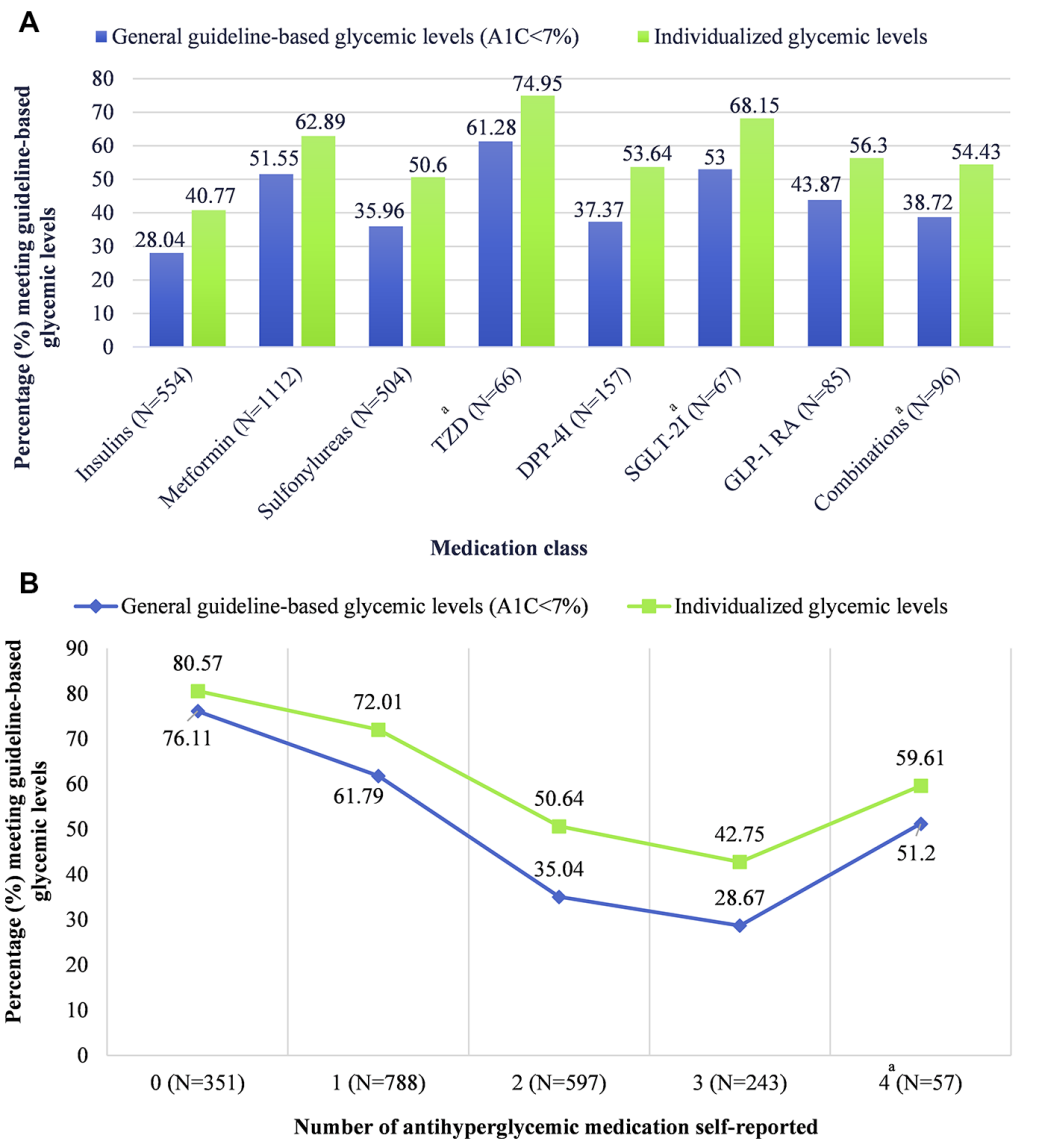
Glycemic levels by **(A)** antihyperglycemic medication class and **(B)** number of antihyperglycemic medications, stratified by general guideline-based glycemic levels versus individualized glycemic levels. Data including measures of uncertainty (95% confidence intervals) are available in table form in Supplementary Table S3. ^a^Caution when interpreting this estimate; estimate reliability may be limited due to the effective denominator sample size being less than 30. Effective sample size: “the sample size divided by the design effect” (33). The estimates for “other” antihyperglycemic medication class (*N* = 27; effective sample size = 10.5) and five antihyperglycemic medications (*N* = 6; effective sample size = 6) were suppressed due to low sample size. TZD, thiazolidinediones; DPP-4i, dipeptidyl peptidase 4 inhibitors; SGLT-2i, sodium-glucose cotransporter-2 inhibitors; GLP-1 RA, glucagon-like peptide 1 receptor agonists.

Glycemic levels differed by the class of antihyperglycemic medication prescribed (Figure 1A; Supplementary Table S3). The lowest percentages of people meeting guideline-based glycemic levels were among people taking insulins (28.04, 95% CI = 20.52–36.58), sulfonylureas (35.96, 95% CI = 28.24–44.24), and dipeptidyl peptidase 4 inhibitors (DPP-4i) (37.37, 95% CI = 21.98–54.88). Results were similar for individualized glycemic levels, but the percentages meeting guideline-based glycemic levels were 40.77% (95% CI = 32.03–49.96), 50.60% (95% CI = 43.61–57.56), and 53.64% (95% CI = 37.21–69.51), respectively. Glycemic levels were significantly different among patients taking different numbers of antihyperglycemic medications (*p* < 0.001). People taking zero medications had the highest percentage for meeting guideline-based glycemic levels (76.11, 95% CI = 70.56–81.08), and the percentage decreased as the number of medications increased (Figure 1B; Supplementary Table S3). Results were similar for individualized glycemic levels. We noted an exception for people taking four antihyperglycemic medications but recommended caution for the reliability of this estimate due to the low sample size.

### 3.3. Predicting guideline-based glycemic levels

The results for the full multivariable logistic regression model with all factors are available in Supplementary Table S4. The logistic regression model predicting meeting guideline-based glycemic levels retained 14 factors after stepwise selection (Table 2). Taking insulin (aOR = 0.16, 95% CI = 0.10–0.26), metformin (aOR = 0.66, 95% CI = 0.46–0.96), sulfonylureas (aOR = 0.36, 95% CI = 0.24–0.55), or combination antihyperglycemic medications (aOR = 0.36, 95% CI = 0.14–0.88) was associated with lower odds of meeting guideline-based glycemic levels compared to not taking each respective medication class. Contextual factors associated with higher odds of meeting guideline-based glycemic levels included not having a family history of diabetes (aOR = 1.43, 95% CI = 1.03–1.98) and reporting healthier diets [“excellent” (aOR = 4.21, 95% CI = 1.92–9.25), “very good” (aOR = 3.07, 95% CI = 1.51–6.21), and “good” (aOR = 1.99, 95% CI = 1.23–3.22) vs. “poor”]. Factors associated with lower odds of meeting guideline-based glycemic levels included male vs. female gender (aOR = 0.61, 95% CI = 0.46–0.80), speaking English and a non-English language vs. speaking English only (aOR = 0.70, 95% CI = 0.50–0.98), marginal versus full food security (aOR = 0.61, 95% CI = 0.44–0.86), less frequent healthcare utilization [none vs. ≥4 times per year (aOR = 0.51, 95% CI = 0.27–0.96) and 2–3 times per year vs. ≥4 times per year (aOR = 0.64, 95% CI = 0.45–0.91)], and being uninsured vs. having insurance to cover prescription drug costs (aOR = 0.51, 95% CI = 0.33–0.79).

**Table 2:** Multivariable logistic regression model with stepwise variable selection for predicting guideline-based glycemic levels (A1C < 7%) among people with diabetes in 2015–2020 NHANES data.

Characteristics	Adjusted odds ratio (95% CI) *N* = 2042
**Fixed patient factors**	
*Gender*	
Male	0.61 (0.46–0.80)^a^
Female	Ref.
*Family history of diabetes*	
Yes	Ref.
No	1.43 (1.03–1.98)^a^
*Acculturation: language spoken at home*	
English only	Ref.
Non-English only	1.02 (0.67–1.57)
English and non-English	0.70 (0.50–0.98)^a^
*Household food security^b^*	
Full food security	Ref.
Marginal food security	0.61 (0.44–0.86)^a^
Low food security	0.89 (0.62–1.26)
Very low food security	0.92 (0.64–1.33)
**Prescribed antihyperglycemic treatment regimens**	
*Class of prescribed antihyperglycemic medication*	
Insulins	
Yes	0.16 (0.10–0.26)^a^
No	Ref.
Metformin	
Yes	0.66 (0.46–0.96)^a^
No	Ref.
Sulfonylureas	
Yes	0.36 (0.24–0.55)^a^
No	Ref.
Combinations	
Yes	0.36 (0.14–0.88)^a^
No	Ref.
**Clinical and psychosocial factors**	
Systolic blood pressure (mmHg)	1.00 (0.99–1.00)
Total cholesterol (mg/dL)	1.00 (0.99–1.00)
**Behaviors**	
*Self-reported healthiness of overall diet*	
Excellent	4.21 (1.92–9.25)^a^
Very good	3.07 (1.51–6.21)^a^
Good	1.99 (1.23–3.22)^a^
Fair	1.58 (1.00–2.51)
Poor	Ref.
**Care processes**	
*Foot exam from doctor within past year*	
Yes	Ref.
No	1.29 (0.98–1.70)
**Patient-physician-system interactions**	
*Frequency of healthcare use over past year*	
≥4 times	Ref.
2 to 3 times	0.64 (0.45–0.91)^a^
Once	0.61 (0.29–1.27)
None	0.51 (0.27–0.96)^a^
*Out-of-pocket prescription costs covered by insurance*	
Insurance does cover prescriptions	Ref.
Insurance does not cover prescription	1.16 (0.63–2.12)
Uninsured	0.51 (0.33–0.79)^a^

C-statistic for the parsimonious model identified through stepwise selection was 0.76. The stepwise selection approach used a conservative *p*-value of 0.10 for variables entry and exit during model building. ^a^Indicates significant result, *p*-value < 0.05. ^b^NHANES assessed survey respondents’ food security using the United States Food Security Survey Module questions. NHANES created the categories of “full food security,” “marginal food security,” “low food security,” and “very low food security” based on the respondents’ number of “affirmative responses” (28).

### 3.4. Sensitivity analysis: predicting individualized glycemic levels

The results for the full multivariable logistic regression model with all factors are available in Supplementary Table S5. The logistic regression model predicting meeting individualized glycemic levels retained 12 factors after stepwise selection (Table 3). Compared to the results above, results were similar for gender, insulin, sulfonylureas, and no insurance for prescription drug costs. Contextual factors associated with higher odds of meeting individualized glycemic levels included a lower diastolic blood pressure (aOR = 0.99, 95% CI = 0.98–1.00), a higher number of chronic conditions (aOR = 1.32, 95% CI = 1.07–1.63), and former versus never smoker (aOR = 1.30, 95% CI = 1.03–1.63). Factors associated with lower odds of meeting individualized glycemic levels included non-White race/ethnicity (Hispanic race/ethnicity (aOR = 0.34, 95% CI = 0.18–0.65), non-Hispanic Black race/ethnicity (aOR = 0.63, 95% CI = 0.44–0.90), and non-Hispanic Asian race/ethnicity (aOR = 0.50, 95% CI = 0.25–0.98) vs. non-Hispanic White/other race/ethnicity), widowed/divorced/separated versus married/living with a partner (aOR = 0.67, 95% CI = 0.50–0.89), and insurance coverage type (private insurance (aOR = 0.42, 95% CI = 0.22–0.80), Medicaid (aOR = 0.36, 95% CI = 0.18–0.74), and other insurance (aOR = 0.47, 95% CI = 0.26–0.86) vs. multiple plans).

**Table 3:** Sensitivity analysis: Multivariable logistic regression model with stepwise variable selection for predicting individualized glycemic levels among people with diabetes in 2015–2020 NHANES data.

Characteristics	Adjusted Odds Ratio (95% CI) *N* = 2042
**Fixed patient factors**	
*Race/ethnicity*	
Non-Hispanic White/other	Ref.
Hispanic	0.34 (0.18–0.65)^a^
Non-Hispanic Black	0.63 (0.44–0.90)^a^
Non-Hispanic Asian	0.50 (0.25–0.98)^a^
*Gender*	
Male	0.69 (0.54–0.88)^a^
Female	Ref.
*Marital status*	
Married/living with partner	Ref.
Widowed/divorced/separated	0.67 (0.50–0.89)^a^
Never married	1.29 (0.80–2.10)
*Acculturation: language spoken at home*	
English only	Ref.
Non-English only	1.69 (0.96–2.96)
English and non-English	1.29 (0.71–2.33)
**Prescribed antihyperglycemic treatment regimens**	
*Class of prescribed antihyperglycemic medication*	
*Insulins*	
Yes	0.17 (0.11–0.27)^a^
No	Ref.
*Sulfonylureas*	
Yes	0.35 (0.24–0.51)^a^
No	Ref.
**Clinical and psychosocial factors**	
Body mass index (kg/m^2^)	0.98 (0.96–1.00)
Diastolic blood pressure (mmHg)	0.99 (0.98–1.00)^a^
Total number of chronic conditions	1.32 (1.07–1.63)^a^
**Behaviors**	
*Smoking status*	
Never smoked	Ref.
Former smoker	1.30 (1.03–1.63)^a^
Current smoker	0.90 (0.62–1.31)
**Patient-physician-system interactions**	
*Insurance coverage type*	
Multiple plans	Ref.
Private insurance	0.42 (0.22–0.80)^a^
Medicare	1.11 (0.73–1.70)
Medicaid	0.36 (0.18–0.74)^a^
Others	0.47 (0.26–0.86)^a^
Uninsured	1.66 (0.47–5.89)
*Out-of-pocket prescription costs covered by insurance*	
Insurance does cover prescriptions	Ref.
Insurance does not cover prescription	1.11 (0.60–2.08)
Uninsured	0.16 (0.04–0.62)^a^

C-statistic for the parsimonious model identified through stepwise selection was 0.77. The stepwise selection approach used a conservative *p*-value of 0.10 for variables entry and exit during model building. ^a^Indicates significant result, *p*-value < 0.05.

## 4. Discussion

Using representative national survey data from 2015 to March 2020, an estimated 52% of people with diabetes in the United States met guideline-based glycemic levels (A1C < 7%); thus, nearly half of the population lived with high glycemic levels. This estimate is similar to other study findings from 1998 to 2018 (10, 11, 29). Meeting guideline-based glycemic levels was associated with the class of prescribed antihyperglycemic medications and various contextual factors, including gender, language spoken, food security, the healthiness of diet, healthcare use, and insurance coverage, among others. Our study adds timely, population-based estimates that can inform national efforts to optimize diabetes management and highlights the differences in findings when glycemic levels are categorized using general guideline recommendations vs. an individualized approach. Finally, the multitude of contextual factors included while studying glycemic levels provides adjusted estimates controlling for influential factors associated with glycemic levels.

Our results for the association between meeting guideline-based glycemic levels and prescribed antihyperglycemic medications are aligned with results from prior work. A1C differs based on the class of antihyperglycemic medications (12, 13). Like previous work, we found insulin use was associated with lower odds of meeting guideline-based glycemic levels than those with no insulin use. In contrast to prior work (12), we found metformin use vs. no use associated with lower odds of meeting guideline-based glycemic levels. Our adjusted analysis provides new evidence for the association of medication class (i.e., insulin, metformin, sulfonylurea, and combinations) after adjusting for covariates. A1C was associated with the number of prescribed antihyperglycemic medications (10, 12). People with A1C ≥ 7% were more likely to use two or three antihyperglycemic medications than those with A1C < 7% (10). Our findings quantify the decreasing prevalence of A1C < 7% as the number of antihyperglycemic medications increases. This finding is likely explained by patients taking zero or one medication having shorter diabetes duration or less severe disease status than those taking two or more medications (24, 30). These patients were also likely treated with lifestyle modifications, where we found that patients taking zero medications reported healthier diets compared to those taking medications, although the association was insignificant. The number of antihyperglycemic medications was significantly associated with glycemic levels in the unadjusted analyses but not in the adjusted analyses. We speculate that this occurred after adjusting for factors related to diabetes severity (e.g., duration of diabetes or number of chronic conditions) and the binary variables for antihyperglycemic medication classes (taking vs. not taking medication in each respective class).

Contextual factors outside of antihyperglycemic medication use were also significantly associated with glycemic levels, such as gender, language spoken, food security, and the healthiness of diet. The TRIAD study previously found females were more susceptible to “poor control” in diabetes (16). However, male gender was associated with lower odds of meeting guideline-based glycemic levels, consistent with the lower rate of glycemic control among males vs. females diagnosed with diabetes from a previous nationally representative study (22). Respondents speaking English and non-English languages vs. only English were less likely to meet guideline-based glycemic levels. Additionally, marginal food security lowered the odds of meeting guideline-based glycemic levels by about 40% compared to full food security, and healthier diets were associated with meeting guideline-based glycemic levels. These findings support the potential influence of social determinants of health (SDOH) on glycemic levels and call for continued research on SDOH in populations with diabetes (31). The association between food security/diet and high glycemic levels also supports policies to improve access to healthy foods in the United States to promote disease control in diabetes.

The comprehensive inclusion of contextual factors from the TRIAD conceptual model is a strength of this study. We capitalized on the availability of individuals’ behavioral and lifestyle (e.g., diet, physical activity, diabetes self-management behaviors) and psychosocial factors (e.g., sleep, depressive symptoms) from the NHANES data. Prior NHANES studies have not holistically analyzed these behavioral, lifestyle, psychosocial, and antihyperglycemic medication use factors (10, 14). Thus, our consideration of multiple contextual factors in understanding the complexities of glycemic levels is innovative compared to a siloed approach (32). Our study expands the evidence base to produce population-based estimates for glycemic levels among people with diabetes in the United States.

The marked difference in results when operationalizing guideline-based glycemic levels using the general cut-off of A1C < 7% versus an individualized cut-off is an interesting discovery. Approximately 63% of adults met individualized A1C goals accounting for age and complications. The factors predicting individualized glycemic levels also differed from those predicting guideline-based glycemic levels (A1C < 7%). When examining individualized glycemic levels, race/ethnicity, marital status, chronic conditions, smoking status, and insurance type emerged as significantly associated factors. These differences demonstrate the importance of determining criteria for operationalizing guideline-based glycemic levels in research and clinical practice. Findings revealed that non-White racial/ethnic groups (Hispanic, non-Hispanic Black, and non-Hispanic Asian) were significantly less likely to meet individualized glycemic levels than non-Hispanic White races/ethnicities. This finding may inform the current health disparities in diabetes management. The number of chronic conditions was also positively associated with meeting individualized glycemic levels. Chronic conditions are crucial in clinical practice to determine a person’s individualized A1C goal following the ADA guidelines (8).

### 4.1. Limitations

Several limitations need to be acknowledged. First, the NHANES data is mainly self-reported and may be influenced by recall bias, social desirability bias, and perhaps selection bias. Second, we could not capture potentially important variables or confounders not included in the NHANES data, such as quality of care, medication adherence, and health literacy. Third, the cross-sectional nature limited our ability to infer causality, temporal sequence, or change over time. Fourth, the NHANES data cannot differentiate between type 1 and type 2 diabetes, so we can only infer findings for the general adult population with diabetes. While we could assume that most people (90–95%) were diagnosed with type 2 diabetes based on the nationally representative nature of the data source (11), we chose not to treat this population as type 2 diabetes only to avoid misclassification bias for those people diagnosed with type 1 diabetes. Finally, the complex sampling design of the NHANES data led to the need for caution when interpreting the reliability of estimates. The reliability of estimated probabilities may be limited when low denominator sample sizes are present, and we flagged these instances to caution readers’ interpretation. Despite these limitations, a major strength of our study findings is the high external validity to the general adult population with diabetes across the United States based on the nationally representative nature of the data source.

## 5. Conclusion

Diabetes management remained a continued public health concern in the United States from 2015-March 2020, when only 52% of people with diabetes met guideline-based glycemic levels. However, considering age and chronic conditions in identifying individualized glycemic goals revealed that 63% of people with diabetes met individualized glycemic goals. Meeting guideline-based glycemic levels was associated with the antihyperglycemic medication class used and various contextual factors. The contextual factors of healthier diet and no family history of diabetes were associated with higher odds of meeting guideline-based glycemic levels, while male gender, speaking English and non-English languages, marginal household food security, less frequent healthcare utilization, and lack of health insurance were associated with lower odds. These identified factors could inform national public health strategies for diabetes management.

## Data availability statement

Publicly available datasets were analyzed in this study. This data can be found at: https://wwwn.cdc.gov/nchs/nhanes/Default.aspx.

## Ethics statement

The studies involving human participants were reviewed and approved by the Auburn University Institutional Review Board through an exempt application. Written informed consent for participation was not required for this study in accordance with the national legislation and the institutional requirements.

## Author contributions

CM and CC contributed to the conception and design of the study. All authors contributed to the study protocol. CM processed the data, performed the statistical analysis, and wrote the first draft of the manuscript. All authors contributed to the article and approved the submitted version.

## Funding

The PhRMA Foundation funded this study through the Pre-Doctoral Fellowship in Health Outcomes Research. CM was previously supported by the American Foundation for Pharmaceutical Education.

## Conflict of interest

W-HL-C has received research funding from Merck Sharp & Dohme Corp and Bristol Myers Squibb. JK is the 2023 Immediate Past President of ADCES (expense stipend), and she received grant funding from the Alabama Department of Public Health to conduct an external evaluation of State support programs for DSMES and DPP.

The remaining authors declare that the research was conducted in the absence of any commercial or financial relationships that could be construed as a potential conflict of interest.

## Publisher’s note

All claims expressed in this article are solely those of the authors and do not necessarily represent those of their affiliated organizations, or those of the publisher, the editors and the reviewers. Any product that may be evaluated in this article, or claim that may be made by its manufacturer, is not guaranteed or endorsed by the publisher.
