# Supplementary material for: Medication use and contextual factors associated with meeting guideline-based glycemic levels in diabetes among a nationally representative sample
Source: Front Med (Lausanne). 2023 May 31;10:1158454. doi: 10.3389/fmed.2023.1158454 (PMC10264805; doi:10.3389/fmed.2023.1158454)
Supplement: Supplementary file 1 [file Table_1.PDF]

Supplementary Material

Medication use and contextual factors associated with meeting guideline-based glycemic levels in diabetes among a nationally representative sample

McDaniel CC, Lo-Ciganic WH, Garza KB, Kavookjian J, Fox BI, Chou C*

*** Correspondence:** Chiahung Chou: czc0109@auburn.edu

1 Supplementary Tables

eTable1. Contextual Factors in Studying Glycemic Levels

eTable2. Antihyperglycemic Medication Classes

eTable3. Data Table for Glycemic Levels by Antihyperglycemic Medication Class and Number of Antihyperglycemic Medications, Stratified by General Guideline-based Glycemic Levels vs. Individualized Glycemic Levels

eTable4. Full Model for Multivariable Logistic Regression Predicting Guideline-based Glycemic Levels (A1C<7%) among People with Diabetes in 2015-2020 NHANES

eTable5. Sensitivity Analysis: Full Model for Multivariable Logistic Regression Predicting Individualized Glycemic Levels among People with Diabetes in 2015-2020 NHANES

eTable1. Contextual Factors in Studying Glycemic Levels

Contextual Factor Category[a]	Contextual Factor	Data Type
Fixed patient factors	Race/Ethnicity	Nominal
	Age	Continuous
	Gender	Nominal
	Family history of diabetes	Nominal
	Family monthly poverty level index	Ordinal
	Marital status	Nominal
	Education level	Ordinal
	Occupation type	Nominal
	Acculturation: Language spoken at home	Nominal
	Household food security	Ordinal
Clinical and psychosocial factors	Depressive symptoms (Total score from Patient Health Questionnaire; PHQ-9 (1))	Continuous
	Duration of diabetes in years	Continuous
	Body mass index (kg/m^2)	Continuous
	Average systolic blood pressure (mmHg)	Continuous
	Average diastolic blood pressure (mmHg)	Continuous
	Total cholesterol (mg/dL)	Continuous
	Total number of chronic conditions (present=1 or absent=0): Asthma, arthritis, congestive heart failure, coronary heart disease, angina, myocardial infarction, stroke, thyroid condition, liver condition, cancer, and chronic obstructive pulmonary disorder or other related conditions (emphysema or chronic bronchitis)	Continuous
	Average sleep hours per night during weekdays	Continuous
Behaviors	Diabetes self-management: Frequency of blood glucose self-monitoring	Ordinal
	Diabetes self-management: Feet self-monitoring	Ordinal
	Healthiness of overall diet	Ordinal
	Physical activity: Metabolic equivalents (METs) (2) per week	Ordinal
	Smoking status	Nominal
Care processes	A1C test within past year	Nominal
	Foot exam from doctor within past year	Nominal
	Eye exam (dilated pupils)	Nominal
Patient-physician-system interactions	Routine place for healthcare use	Nominal
	Frequency of healthcare use over past year	Ordinal
	Insurance coverage type	Nominal
	Out-of-pocket prescription costs covered by insurance	Nominal

[a]Groupings within contextual factor categories were informed by the TRIAD conceptual model (3).

eTable2. Antihyperglycemic Medication Classes

Medication Class[a]	Medication Name: Multum's Lexicon Generic Drug Code[a]	Route of Admin. (4)	Medication Characteristics (4)
Biguanides	Metformin: d03807	Oral	Risk for gastrointestinal side effects; may help with minor weight loss
Sulfonylureas	Acetohexamide: d00162<br>chlorpropamide: d00042<br>Gliclazide: h00034<br>Glimepiride: d03864<br>Glipizide: d00246<br>Glyburide: d00248<br>Tolazamide: d00393<br>Tolbutamide: d00394	Oral	Risk for hypoglycemia; may cause weight gain
TZD	Pioglitazone: d04442<br>Rosiglitazone: d04434<br>Troglitazone: d04122	Oral	May cause weight gain
DPP-4i	Alogliptin: d07963<br>Linagliptin: d07767<br>Simvastatin/sitagliptin: d07805<br>Sitagliptin: d05896<br>Saxagliptin: d07467	Oral	Need for modification of renal dose (except linagliptin)
SGLT-2i	Canagliflozin: d08080<br>Dapagliflozin: d07928<br>Empagliflozin: d08275<br>Ertugliflozin: d08689	Oral	May help with weight loss; cardiovascular and renal benefits
GLP-1 RA	Albiglutide: d08251<br>Dulaglutide: d08290<br>Exenatide: d05529<br>Liraglutide: d07466<br>Semaglutide: d08688	Subcutaneous injection (semaglutide available as oral)	May help with weight loss; cardiovascular and renal benefits
Insulins	Insulin: d00262<br>Insulin aspart: d04697<br>Insulin aspart/insulin aspart protamine: d04839<br>Insulin degludec: d08054<br>Insulin detemir: d05436<br>Insulin glargine: d04538<br>Insulin glulisine: d05278<br>Insulin inhalation rapid acting: d05765<br>Insulin isophane (NPH): d04370<br>Insulin isophane/insulin regular: d04374<br>Insulin lispro: d04373<br>Insulin lispro/insulin lispro protamine: d04510	Subcutaneous injection	Risk for hypoglycemia; may cause weight gain

**Medication Class**[a]	**Medication Name: Multum's Lexicon Generic Drug Code**[a]	**Route of Admin. (4)**	**Medication Characteristics (4)**
	Insulin regular: d04369<br>Insulin zinc: d04371<br>Insulin zinc extended: d04372		
Combinations	Alogliptin/pioglitazone: d07965<br>Canagliflozin/metformin: d08280<br>Chlorpromamide/phenformin: h00013<br>Dapagliflozin/metformin: d08245<br>Empagliflozin/linagliptin: d08344<br>Empagliflozin/metformin: d08384<br>Ertugliflozin/sitagliptin: d08692<br>Glimepiride/rosiglitazone: d05674<br>Glipizide/metformin: d04823<br>Glyburide/metformin: d04703<br>Insulin degludec/liraglutide: d08311<br>Insulin glargine/lixisenatide: d08501<br>Linagliptin/metformin: d07825<br>Metformin/pioglitazone: d05635<br>Metformin/rosiglitazone: d04820<br>Metformin/saxagliptin: d07709<br>Metformin/sitagliptin: d06720	Based on individual medications for each combination	Based on individual medications for each combination
Others	<u>α-glucosidase inhibitors</u><br>Acarbose: d03846<br>Miglitol: d04110<br><u>Antidiabetic agents – unspecified:</u> c00099<br><u>Bile acid sequestrants</u><br>Colesevelam: d04695<br>Colestipol: d00744<br>Cholestyramine: d00193<br><u>Meglitinides</u><br>Repaglinide: d04267<br>Nateglinide: d04743	Oral	

Abbreviations: TZD=thiazolidinediones, DPP-4i=dipeptidyl peptidase 4 inhibitors, SGLT-2i=sodium-glucose cotransporter-2 inhibitors, GLP-1 RA=glucagon-like peptide 1 receptor agonists.

[a]The NHANES drug data was classified using Multum's Lexicon database from Cerner Multum, Inc. (5). Multum's Lexicon first level classification: metabolic agents (code=358); Multum's Lexicon second level classification: antidiabetic agents (code=99); Multum's Lexicon third level classification: sulfonylureas (code=213), biguanides (code=214), insulin (code=215), α-glucosidase inhibitors (code=216), thiazolidinediones (code=271), meglitinides (code=282), antidiabetic combinations (code=314), DPP-4i (code=371), amylin analogs (code=372), GLP-1 RA (code=373), SGLT-2i (code=458) (5, 6). Bile acid sequestrants were also included using Multum's Lexicon third level classification code 252 (5, 6). The medication names included in the table are based on those available in the study sample from the 2015-2020 March Pre-pandemic NHANES database (6). Pramlintide (Multum's Lexicon Generic Drug Code: d05488) and bromocriptine (Multum's Lexicon Generic Drug Code: d00178) were originally included in the drug classification as "others"; however, no records were self-reported for these medications among the study sample.

eTable3. Data Table for Glycemic Levels by Antihyperglycemic Medication Class and Number of Antihyperglycemic Medications, Stratified by General Guideline-based Glycemic Levels vs. Individualized Glycemic Levels

Medication Class	Percentage Meeting Guideline-based Glycemic Levels (A1C<7%) Weighted % (95% CI); N	Percentage Not Meeting Guideline-based Glycemic Levels (A1C≥7%) Weighted % (95% CI); N	p-value
Insulins	28.04 (20.52-36.58); N=116	71.96 (63.42-79.48); N=438	<.001
Metformin	51.55 (45.64-57.43); N=507	48.45 (42.57-54.36); N=605	.864
Sulfonylureas	35.96 (28.24-44.24); N=171	64.04 (55.76-71.76); N=333	<.001
TZD[a]	61.28 (39.11-80.48); N=34	38.72 (19.52-60.89); N=32	.383
DPP-4i	37.37 (21.98-54.88); N=53	62.63 (45.12-78.02); N=104	.066
SGLT-2i[a]	53.00 (30.52-74.61); N=22	47.01 (25.39-69.48); N=45	.903
GLP-1 RA	43.87 (23.39-66.01); N=28	56.13 (33.99-76.61); N=57	.387
Combinations[a]	38.72 (22.11-57.50); N=37	61.28 (42.50-77.89); N=59	.140
Medication Class	Percentage Meeting Individualized Glycemic Levels Weighted % (95% CI); N	Percentage Not Meeting Individualized Glycemic Levels Weighted % (95% CI); N	p-value
Insulins	40.77 (32.03-49.96); N=188	59.23 (50.04-67.97); N=366	<.001
Metformin	62.89 (58.05-67.55); N=621	37.11 (32.45-41.95); N=491	.885
Sulfonylureas	50.60 (43.61-57.56); N=239	49.40 (42.44-56.39); N=265	<.001
TZD[a]	74.95 (55.39-89.11); N=43	25.05 (10.89-44.61); N=23	.176
DPP-4i	53.64 (37.21-69.51); N=79	46.36 (30.49-62.79); N=78	.198
SGLT-2i[a]	68.15 (48.65-83.89); N=30	31.85 (16.11-51.35); N=37	.532
GLP-1 RA	56.30 (35.32-75.75); N=39	43.70 (24.25-64.68); N=46	.422
Combinations[a]	54.43 (39.39-68.91); N=52	45.57 (31.09-60.61); N=44	.195
Number of Antihyperglycemic Medications	Percentage Meeting Guideline-based Glycemic Levels (A1C<7%) Weighted % (95% CI); N	Percentage Not Meeting Guideline-based Glycemic Levels (A1C≥7%) Weighted % (95% CI); N	p-value
Zero	76.11 (70.56-81.08); N=253	23.89 (18.92-29.44); N=98	<.001
One	61.79 (56.06-67.28); N=462	38.21 (32.72-43.94); N=326	
Two	35.04 (28.01-42.59); N=181	64.96 (57.41-71.99); N=416	
Three	28.67 (20.13-38.49); N=56	71.33 (61.51-79.87); N=187	
Four[a]	51.20 (29.69-72.39); N=16	48.80 (27.61-70.31); N=41	
Number of Antihyperglycemic Medications	Percentage Meeting Individualized Glycemic Levels Weighted % (95% CI); N	Percentage Not Meeting Individualized Glycemic Levels Weighted % (95% CI); N	p-value
Zero	80.57 (75.27-85.17); N=270	19.43 (14.83-24.73); N=81	<.001
One	72.01 (67.76-75.99); N=527	27.99 (24.01-32.24); N=261	
Two	50.64 (42.65-58.61); N=264	49.36 (41.39-57.35); N=333	
Three	42.75 (32.73-53.23); N=91	57.25 (46.77-67.27); N=152	
Four[a]	59.61 (39.02-77.99); N=24	40.39 (22.01-60.98); N=33	

[a]Caution when interpreting this estimate; estimate reliability may be limited due to the effective denominator sample size being less than 30. Effective sample size: "the sample size divided by the design effect" (7). The estimates for "other" medication class (N=27; effective sample size=10.5) and five antihyperglycemic medications (N=6; effective sample size=6) were suppressed due to low sample size. Abbreviations: TZD=thiazolidinediones, DPP-4i=dipeptidyl peptidase 4 inhibitors, SGLT-2i=sodium-glucose cotransporter-2 inhibitors, GLP-1 RA=glucagon-like peptide 1 receptor agonists.

eTable4. Full Model for Multivariable Logistic Regression Predicting Guideline-based Glycemic Levels (A1C<7%) among People with Diabetes in 2015-2020 NHANES

Characteristics	Adjusted Odds Ratio (95% CI) N=2042
Fixed Patient Factors	
Age	0.99 (0.97-1.01)
Race/Ethnicity	
Non-Hispanic White/other	Ref.
Hispanic	0.54 (0.30-0.96)[a]
Non-Hispanic Black	0.84 (0.57-1.25)
Non-Hispanic Asian	0.49 (0.26-0.93)[a]
Gender	
Male	0.53 (0.39-0.70)[a]
Female	Ref.
Family history of diabetes	
Yes	Ref.
No	1.36 (0.99-1.86)
Family monthly poverty level index	
≤1.30	1.04 (0.71-1.53)
1.30 < index ≤ 1.85	1.08 (0.73-1.59)
>1.85	Ref.
Marital status	
Married/living with partner	Ref.
Widowed/divorced/separated	0.74 (0.56-0.99)[a]
Never married	1.25 (0.80-1.95)
Education level	
Less than high school	0.90 (0.54-1.49)
High school graduate/GED	0.81 (0.56-1.17)
Some college/associate degree	1.02 (0.67-1.55)
College graduate or above	Ref.
Occupation type	
Looking for work with a job/business but not at work	1.11 (0.60-2.06)
Not working at a job/business	1.13 (0.77-1.66)
Working at a job/business for work	Ref.
Acculturation: Language spoken at home	
English only	Ref.
Non-English only	1.78 (1.04-3.06)[a]
English and non-English	1.20 (0.71-2.03)
Household food security	
Full food security	Ref.
Marginal food security	0.63 (0.44-0.90)[a]
Low food security	0.85 (0.55-1.33)
Very low food security	0.87 (0.58-1.31)
Prescribed Antihyperglycemic Treatment Regimens	

Characteristics	Adjusted Odds Ratio (95% CI) N=2042
Class of prescribed antihyperglycemic medication	
Insulins	
Yes	0.25 (0.09-0.72)[a]
No	Ref.
Metformin	
Yes	0.89 (0.43-1.86)
No	Ref.
Sulfonylureas	
Yes	0.50 (0.21-1.21)
No	Ref.
TZD	
Yes	2.00 (0.68-5.88)
No	Ref.
DPP-4i	
Yes	0.71 (0.27-1.90)
No	Ref.
SGLT-2i	
Yes	2.14 (0.59-7.68)
No	Ref.
GLP-1 RA	
Yes	0.93 (0.34-2.56)
No	Ref.
Combinations	
Yes	0.73 (0.16-3.37)
No	Ref.
Others	
Yes	0.81 (0.25-2.61)
No	Ref.
Number of prescribed antihyperglycemic medications (0-5)	0.78 (0.37-1.64)
Clinical and Psychosocial Factors	
Depressive symptoms (PHQ-9 score)	1.02 (0.99-1.05)
Duration of diabetes in years	1.01 (0.99-1.02)
Body mass index (kg/m^2)	0.99 (0.97-1.01)
Systolic blood pressure (mmHg)	1.00 (0.99-1.00)
Diastolic blood pressure (mmHg)	1.00 (0.98-1.01)
Total cholesterol (mg/dL)	1.00 (0.99-1.00)[a]
Average sleep hours per night during weekdays	1.01 (0.93-1.10)
Total number of chronic conditions	1.05 (0.93-1.18)
Behaviors	
Frequency of blood glucose self-monitoring	
Never	Ref.
Multiple times daily	0.64 (0.39-1.07)
Once daily	0.69 (0.42-1.12)

Characteristics	Adjusted Odds Ratio (95% CI)<br>N=2042
Weekly (once or more)	0.75 (0.49-1.14)
Less than weekly	0.88 (0.51-1.51)
Frequency of feet self-monitoring	
Never	Ref.
Daily (once or more)	1.00 (0.60-1.69)
Weekly (once or more)	1.27 (0.82-1.95)
Less than weekly	0.73 (0.36-1.48)
Self-reported healthiness of overall diet	
Excellent	4.73 (2.11-10.60)[a]
Very good	3.44 (1.78-6.65)[a]
Good	2.17 (1.30-3.60)[a]
Fair	1.76 (1.09-2.86)[a]
Poor	Ref.
Smoking status	
Never smoked	Ref.
Former smoker	1.17 (0.85-1.62)
Current smoker	1.15 (0.75-1.76)
Physical activity: MET minutes per week	
Physical activity not reported	0.99 (0.64-1.52)
Does not meet recommendation (<450)	1.14 (0.73-1.78)
Meets recommendation (450 ≤ MET ≤ 750)	1.24 (0.71-2.17)
Exceeds recommendation (>750)	Ref.
Care Processes	
A1C test within past year	
Yes	Ref.
No	1.22 (0.80-1.86)
Foot exam from doctor within past year	
Yes	Ref.
No	1.27 (0.97-1.67)
Most recent eye exam (dilated pupils)	
Within the past year	Ref.
Over one year ago	1.00 (0.67-1.50)
Never	1.37 (0.79-2.36)
Patient-Physician-System Interactions	
Routine place for healthcare use	
Yes	Ref.
No	0.64 (0.30-1.37)
Frequency of healthcare use over past year	
≥4 times	Ref.
2 to 3 times	0.65 (0.46-0.91)[a]
Once	0.66 (0.34-1.27)
None	0.59 (0.27-1.32)

Characteristics	Adjusted Odds Ratio (95% CI)<br>N=2042
Insurance coverage type	
Private insurance	0.68 (0.38-1.24)
Medicare	0.75 (0.42-1.33)
Medicaid	0.41 (0.16-1.06)
Others	0.79 (0.45-1.39)
Multiple plans	Ref.
Uninsured	0.70 (0.10-5.05)
Out-of-pocket prescription costs covered by insurance	
Insurance does cover prescriptions	Ref.
Insurance does not cover prescription	1.15 (0.63-2.11)
Uninsured	0.49 (0.07-3.66)
NHANES cycle	
2015-2016	0.89 (0.59-1.33)
2017-March 2020 Pre-pandemic	Ref.

Abbreviations: TZD=thiazolidinediones, DPP-4i=dipeptidyl peptidase 4 inhibitors, SGLT-2i=sodium-glucose cotransporter-2 inhibitors, GLP-1 RA=glucagon-like peptide 1 receptor agonists, PHQ-9= Patient Health Questionnaire-9, MET=metabolic equivalent.

C-statistic for the full logistic regression model was 0.76.

[a]Indicates significant result, p-value <.05.

eTable5. Sensitivity Analysis: Full Model for Multivariable Logistic Regression Predicting Individualized Glycemic Levels among People with Diabetes in 2015-2020 NHANES

Characteristics	Adjusted Odds Ratio (95% CI) N=2042
Fixed Patient Factors	
Age	1.00 (0.99-1.02)
Race/Ethnicity	
Non-Hispanic White/other	Ref.
Hispanic	0.35 (0.20-0.62)[a]
Non-Hispanic Black	0.63 (0.43-0.92)[a]
Non-Hispanic Asian	0.42 (0.23-0.79)[a]
Gender	
Male	0.62 (0.48-0.80)[a]
Female	Ref.
Family history of diabetes	
Yes	Ref.
No	1.11 (0.82-1.49)
Family monthly poverty level index	
≤1.30	0.92 (0.62-1.35)
1.30 < index ≤ 1.85	0.78 (0.49-1.23)
>1.85	Ref.
Marital status	
Married/living with partner	Ref.
Widowed/divorced/separated	0.66 (0.49-0.88)[a]
Never married	1.34 (0.84-2.13)
Education level	
Less than high school	0.89 (0.51-1.57)
High school graduate/GED	0.82 (0.55-1.23)
Some college/associate degree	0.97 (0.60-1.56)
College graduate or above	Ref.
Occupation type	
Looking for work with a job/business but not at work	0.83 (0.40-1.72)
Not working at a job/business	1.13 (0.73-1.75)
Working at a job/business for work	Ref.
Acculturation: Language spoken at home	
English only	Ref.
Non-English only	1.66 (0.99-2.78)
English and non-English	1.34 (0.78-2.31)
Household food security	
Full food security	Ref.
Marginal food security	0.81 (0.56-1.16)
Low food security	0.72 (0.43-1.18)
Very low food security	0.98 (0.62-1.55)
Prescribed Antihyperglycemic Treatment Regimens	

Characteristics	Adjusted Odds Ratio (95% CI) N=2042
Class of prescribed antihyperglycemic medication	
Insulins	
Yes	0.26 (0.08-0.81)[a]
No	Ref.
Metformin	
Yes	1.07 (0.53-2.16)
No	Ref.
Sulfonylureas	
Yes	0.55 (0.24-1.28)
No	Ref.
TZD	
Yes	2.14 (0.61-7.52)
No	Ref.
DPP-4i	
Yes	0.92 (0.35-2.43)
No	Ref.
SGLT-2i	
Yes	3.11 (0.96-10.15)
No	Ref.
GLP-1 RA	
Yes	1.12 (0.40-3.10)
No	Ref.
Combinations	
Yes	1.15 (0.24-5.62)
No	Ref.
Others	
Yes	1.90 (0.56-6.45)
No	Ref.
Number of prescribed antihyperglycemic medications (0-5)	0.66 (0.31-1.40)
Clinical and Psychosocial Factors	
Depressive symptoms (PHQ-9 score)	1.03 (1.00-1.06)[a]
Duration of diabetes in years	1.00 (0.98-1.01)
Body mass index (kg/m^2)	0.98 (0.96-1.00)
Systolic blood pressure (mmHg)	1.01 (1.00-1.01)
Diastolic blood pressure (mmHg)	0.98 (0.97-1.00)[a]
Total cholesterol (mg/dL)	1.00 (0.99-1.00)
Average sleep hours per night during weekdays	0.99 (0.91-1.08)
Total number of chronic conditions	1.28 (1.06-1.53)[a]
Behaviors	
Frequency of blood glucose self-monitoring	
Never	Ref.
Multiple times daily	0.84 (0.46-1.54)
Once daily	1.09 (0.60-1.98)

Characteristics	Adjusted Odds Ratio (95% CI) N=2042
Weekly (once or more)	0.87 (0.53-1.44)
Less than weekly	0.77 (0.40-1.48)
Frequency of feet self-monitoring	
Never	Ref.
Daily (once or more)	0.81 (0.50-1.30)
Weekly (once or more)	1.00 (0.66-1.51)
Less than weekly	0.75 (0.39-1.43)
Self-reported healthiness of overall diet	
Excellent	2.93 (0.95-9.06)
Very good	2.71 (1.00-7.34)
Good	1.91 (0.79-4.63)
Fair	1.91 (0.83-4.38)
Poor	Ref.
Smoking status	
Never smoked	Ref.
Former smoker	1.34 (1.03-1.74)[a]
Current smoker	0.94 (0.62-1.42)
Physical activity: MET minutes per week	
Physical activity not reported	1.18 (0.83-1.68)
Does not meet recommendation (<450)	1.08 (0.76-1.54)
Meets recommendation (450 ≤ MET ≤ 750)	1.25 (0.78-2.00)
Exceeds recommendation (>750)	Ref.
Care Processes	
A1C test within past year	
Yes	Ref.
No	1.40 (0.89-2.20)
Foot exam from doctor within past year	
Yes	Ref.
No	1.15 (0.81-1.63)
Most recent eye exam (dilated pupils)	
Within the past year	Ref.
Over one year ago	0.85 (0.60-1.21)
Never	1.45 (0.80-2.64)
Patient-Physician-System Interactions	
Routine place for healthcare use	
Yes	Ref.
No	0.80 (0.41-1.54)
Frequency of healthcare use over past year	
≥4 times	Ref.
2 to 3 times	0.83 (0.54-1.26)
Once	0.73 (0.36-1.47)
None	0.60 (0.28-1.30)

Characteristics	Adjusted Odds Ratio (95% CI)<br>N=2042
Insurance coverage type	
Private insurance	0.54 (0.30-0.98)[a]
Medicare	1.12 (0.71-1.79)
Medicaid	0.43 (0.20-0.93)[a]
Others	0.53 (0.30-0.95)[a]
Multiple plans	Ref.
Uninsured	1.86 (0.44-7.92)
Out-of-pocket prescription costs covered by insurance	
Insurance does cover prescriptions	Ref.
Insurance does not cover prescription	1.33 (0.74-2.37)
Uninsured	0.19 (0.04-0.83)[a]
NHANES cycle	
2015-2016	0.83 (0.57-1.21)
2017-March 2020 Pre-pandemic	Ref.

Abbreviations: TZD=thiazolidinediones, DPP-4i=dipeptidyl peptidase 4 inhibitors, SGLT-2i=sodium-glucose cotransporter-2 inhibitors, GLP-1 RA=glucagon-like peptide 1 receptor agonists, PHQ-9= Patient Health Questionnaire-9, MET=metabolic equivalent.

C-statistic for the full logistic regression model predicting individualized glycemic targets was 0.79.

[a]Indicates significant result, p-value <.05.

2 References for Supplementary Materials

1. Kroenke K, Spitzer RL, Williams JB. The PHQ-9: Validity of a brief depression severity measure. J Gen Intern Med (2001) 16:606-13. doi:10.1046/j.1525-1497.2001.016009606.x
2. Haskell WL, Lee IM, Pate RR, Powell KE, Blair SN, Franklin BA, et al. Physical activity and public health: Updated recommendation for adults from the American College of Sports Medicine and the American Heart Association. Med Sci Sports Exerc (2007) 39:1423-34. doi:10.1249/mss.0b013e3180616b27
3. Selby JV, Swain BE, Gerzoff RB, Karter AJ, Waitzfelder BE, Brown AF, et al. Understanding the gap between good processes of diabetes care and poor intermediate outcomes: Translating Research Into Action for Diabetes (TRIAD). Med Care (2007) 45:1144-53. doi:10.1097/MLR.0b013e3181468e79
4. ElSayed NA, Aleppo G, Aroda VR, Bannuru RR, Brown FM, Bruemmer D, et al. 9. Pharmacologic approaches to glycemic treatment: Standards of care in diabetes—2023. Diabetes Care (2022) 46:S140-S57. doi:10.2337/dc23-S009
5. Cerner Multum, Inc. Lexicon Plus® [Internet]. Available from: www.cerner.com/solutions/drug-database.
6. National Health and Nutrition Examination Survey. 1988-2020 Data Documentation, Codebook, and Frequencies: Prescription Medications - Drug Information (RXQ_DRUG). https://wwwn.cdc.gov/Nchs/Nhanes/1999-2000/RXQ_DRUG.htm#Appendix_3:_Multum_Lexicon_Therapeutic_Classification_Scheme_ [Accessed January 16, 2023].
7. Parker JD, Talih M, Malec DJ, Beresovsky V, Carroll M, Gonzalez JF, et al. National Center for Health Statistics data presentation standards for proportions. Vital Health Stat (2017) 2:1-22.
